# Room Temperature Phosphorescence of Chlorine Doped Carbon Nitride Dots

**DOI:** 10.3389/fchem.2022.812602

**Published:** 2022-03-17

**Authors:** Khemnath Patir, Sonit Kumar Gogoi

**Affiliations:** ^1^ Department of Chemistry, Gauhati University, Guwahati, India; ^2^ Department of Applied Science and Humanities, Assam University, Silchar, India

**Keywords:** guanidine hydrochloride, carbon nitride dots, blue fluorescence, room temperature phosphorescence, security encryption

## Abstract

Metal free room temperature phosphorescent materials have been the subject of considerable attention due to their potential applications in optoelectronic devices sensing, and security and safety signage. This study discusses how efficient fluorescent and phosphorescent chlorine doped carbon nitride dots (Cl-CNDs) were prepared by thermal treatment of guanidine hydrochloride. The Cl-CNDs prepared were characterized by field emission scanning electron microscope, dynamic light scattering, PXRD, EDX, Thermo gravimetric analysis, FT-IR, and UV-Visible spectroscopy. The Cl-CNDs exhibit a long phosphorescence lifetime of 657 ms and the phosphorescence quantum yield was found to be 2.32% upon being excited at 360 nm in ambient conditions. Formation of compact coreparticles via condensation along with hydrogen bonding of Cl-CNDs by its functional groups facilitate intersystem crossing and stabilizes the triplet states, favoring room temperature phosphorescence. The cost effective preparation and tunable optical properties of Cl-CNDs may find applications in security encryption and optoelectronic devices.

## Introduction

Room temperature phosphorescence (RTP) materials with efficient luminescence have received a great deal of attention due to their potential applications in photocatalysis, security features, sensing and optoelectronic devices, etc. due to the longer lifetime of the triplet states (Braun and Heeger, 1991; Zhang et al., 2010; Meruga et al., 2012; Gan et al., 2018). Until the recent reports on carbon nanoparticle based organic phosphorescent materials, most of the materials with desirable RTP were metal doped inorganic and organometallic compounds (Chakraborty et al., 2016; Yang and Yan, 2016; Hu et al., 2020; Wei et al., 2021). However, these materials are inevitably expensive with low stability, and were toxic in nature.

Contrary to its metal based counterpart, the RTP phenomenon from organic materials is difficult to come by as it involves a spin-forbidden process of intersystem crossing (ISC) between the singlet and triplet states (Yuan et al., 2010). As a way of resolving these issues, two methodologies have been developed. The first approach aims to facilitate spin-orbit coupling by incorporating heteroatoms/groups such as phosphorus, nitrogen, aromatic carbonyl or heterocycles, heavy atoms, etc. This helps in adjusting the singlet and triplet state energy gaps to promote efficient ISC. Furthermore, it also helps in inducing non-covalent interactions like hydrogen and halogen bonding, etc. among the phosphorescing species, thus increasing the efficiency of the RTP process (Li et al., 2016; Gao et al., 2018; Long et al., 2018; Patir and Gogoi, 2019). In the second method, RTP is achieved by embedding the luminescent species in solid matrices such as polymer, silica, filter paper, crystals, etc., which again introduces weak interactions suppressing the non-radiative transitions, stabilizes the triplet excited states, and creates a good oxygen barrier in the composite systems (Zhao et al., 2011; Li et al., 2016; Chen et al., 2017).

Carbon nitride quantum dots (CNQD) as an emerging class of luminescent materials have stimulated extensive research in various fields, including bioimaging, chemical sensing, fluorescent marking, optoelectronic devices, etc. due to their excellent optical properties, simple synthesis process, low toxicity, and easy miscibility in different solvents (Liu et al., 2012; Song et al., 2016; Wang et al., 2016; Wang et al., 2017; Patir and Gogoi, 2018). Even though the fluorescent properties of CNQD have been extensively studied, its phosphorescence phenomenon and related applications remain lesser explored. On the other hand, similar luminescent carbon-based nanoparticles, carbon dots (CDs) have been extensively studied as phosphorescent materials in various matrix such as polyvinylalcohol (PVA), urea/biuret, polyurethane, potash alum, silica gel, double layer hydroxides, boric acid, etc. (Deng et al., 2013; Ding et al., 2015; Li et al., 2016; Tan et al., 2016; Bai et al., 2017; Joseph and Anappara, 2017; Li et al., 2019).

The quest for a brighter long-lasting afterglow drives the exploration of RTP materials. At the same time, easy and economic synthetic routes from benign precursors are much desired. Among the two strategies mentioned above, the heteroatom introduction strategy seems advantageous at present as it not only induces phosphorescence by providing mechanical rigidity and oxygen barrier but also the energy tunability of the electronic states; thus allowing control over the emission color of the phosphor.

The present study reports on a facile one step thermal method for synthesizing phosphorescent chlorine doped carbon nitride dots (Cl-CNDs) using low-cost guanidine hydrochloride as the precursor. The Cl-CNDs is highly miscible in water giving a clear dispersion with violet-blue fluorescence under UV irradiation. In powder form, Cl-CNDs show green RTP with an average lifetime of 657 ms along with blue fluorescence. Based on the Cl-CNDs phosphorescence, it is efficiently applied in security marking. Guanidine hydrochloride is a commonly used precursor for the synthesis of bulk graphitic carbon nitride and carbon nitride quantum dots due to its easy availability, low cost, and benign nature (Tang et al., 2013; Tang et al., 2014).

## Experimental Section

### Materials

For this experiment, guanidine hydrochloride and ethanol were purchased from Merck Specialties Private Limited. Transparent liquid paper gum was bought from a local market. Double distilled water was used throughout the experiments.

### Methods

#### Synthesis of phosphorescent chlorine doped carbon nitride dots (Cl-CNDs)

In an optimized synthetic process, 1 g guanidine hydrochloride is transferred to silica crucible with lid and heated at 260°C in an oven from Biocraft Scientific System Pvt. Ltd. for 6 h and allowed to cool to room temperature: a yellowish solid product (Cl-CNDs) is obtained. The solid product so obtained is washed several times with 10 mL portions of ethanol and filtered through Whatman 40 filter paper several times. The washed and dried powder Cl-CNDs is then used for further experiments.

#### Preparation of Cl-CNDs phosphorescent ink

0.01 g of Cl-CNDs is thoroughly mixed to a transparent liquid paper gum and characters ‘CHEM’is written on non-reflecting filter paper.

### Characterization

The scanning electron microscopy (SEM) images of the sample are recorded with a field emission scanning electron microscope (FESEM), Carl Zeiss, Gemini, Model-Sigma 300. The elemental composition of the sample is determined with EDX spectroscopy (EDAX system attached with FESEM) and Thermo Scientific Flash 2000 elemental analyzer. The particle size of the Cl-CNDs is measured through dynamic light scattering (DLS) with a zeta seizer, Nano series Nano-ZS90 (Malvern, United Kingdom). UV 1800 spectrophotometer (SHIMADZU, Japan) is used for recording the UV-visible absorption of the sample, in the range from 200 to 800 nm. The powder X-ray diffraction (PXRD) pattern of the sample is recorded from 5° to 80° 2θ with a scanning speed of 10° per minute in a Rigaku Ultima IV instrument with CuK_α_ radiation (*λ* = 1.54Ǻ). Fourier transform infrared (FT-IR) spectrum of the sample is recorded in SHIMADZU IR Affinity equipment in the range from 400 to 4,000 cm^−1^ in KBr pellets. Thermo gravimetric analysis (TGA) is carried out in a METTLER TOLEDO STAR thermal analysis system. Photoluminescence measurements (fluorescence and phosphorescence spectra) and phosphorescence lifetime decay profile is measured with Hitachi F-7000 spectrophotometer. The time resolved photoluminescence (TRPL) decay of the sample is recorded using the FSP920 Edinburgh Instrument. The chromaticity diagram along with CIE coordinates of the samples are analyzed with OSRAM color calculator software. The phosphorescence quantum yield measurement of the sample is determined in a Horiba Fluoromax-4CP equipped with an integrating sphere.

## Results and Discussion

**SCHEME 1:**
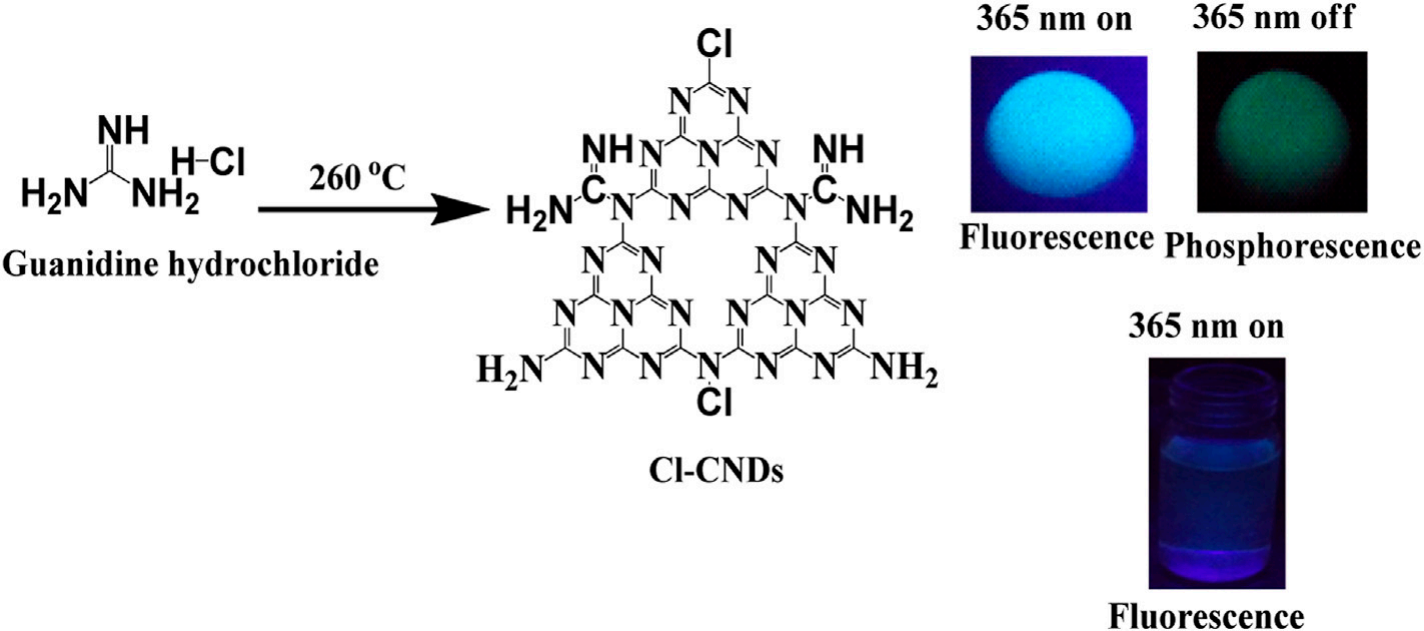
Synthetic scheme of Cl-CNDs and its optical properties.

**FIGURE 1:**
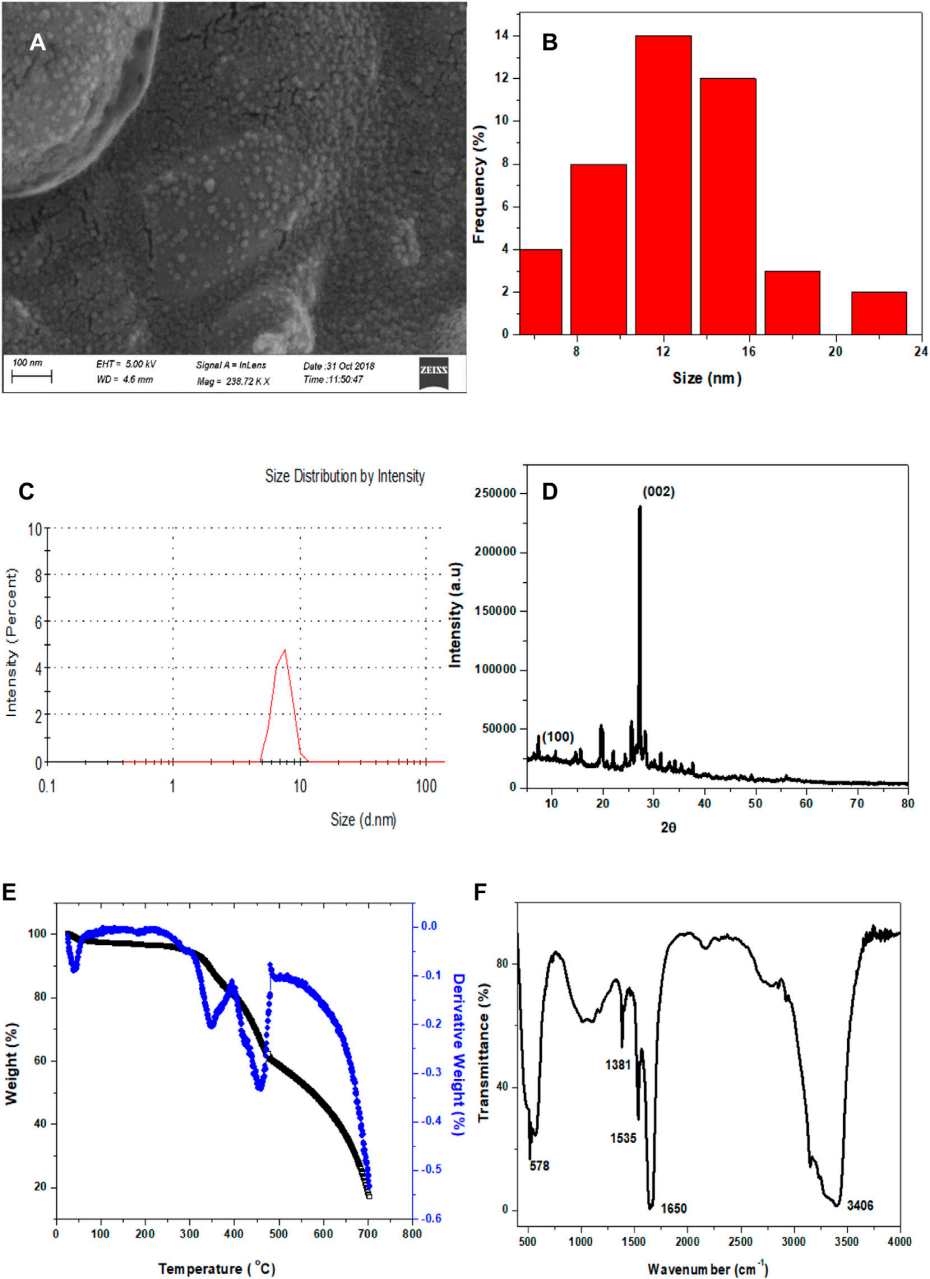
**(A)** FESEM image of Cl-CNDs. **(B)** Particle size distribution from the FESEM image, calculated by using ImageJ software. **(C)** DLS of Cl-CNDs. **(D)** PXRD pattren of Cl-CNDs. **(E)** TGA (black line) and DTG (blue line) of Cl-CNDs. **(F)** FT-IR spectrum of Cl-CNDs.

The phosphorescent Cl-CNDs is directly obtained by heating guanidine hydrochloride at 260 °C in an oven, as illustrated in Scheme 1. The morphology and size of Cl-CNDs are analyzed by FESEM and DLS, Figure 1. The FESEM image of Cl-CNDs shows the uniform particle distribution with sizes below 30 nm, Figure 1A.

The particle distribution of Cl-CNDs from the FESEM image was found to be between 6 and 22 nm, with an average diameter of about 12 nm, Figure 1B. The DLS analysis of Cl-CNDs shows its particle size was 8 nm, which is in agreement with the FESEM results, Figure 1C. The PXRD pattern of Cl-CNDs has peaks at 14.87^°^ 2θ (100) and 27.39^°^ 2θ (200) due to the triazine repeating unit and layered graphitic structure, respectively, which is similar to previous reports, Figure 1D (Deng et al., 2013; Tang et al., 2013; Yuan et al., 2019).

The TGA and DTG curves of Cl-CNDs are shown in Figure 1E. A small weight loss was observed at 38 °C due to the removal of gaseous matter, which may be adsorbed on the Cl-CNDs. The weight losses at approximately 347 and 456 °C are due to the further condensation processes of Cl-CNDs with the elimination of gases like NH_3_ and HCl leading to the formation of bulk carbon nitride (Tang et al., 2013; Rangel et al., 2015).

The FT-IR spectrum of Cl-CNDs, Figure 1F, provides information about the different functional groups present in Cl-CNDs. The broad peak observed at 3,406 cm^−1^ is assigned to–NH/OHstretching vibration while peaks at 1,650 cm^−1^ and 1,381 cm^−1^ are due to C=N and C-N stretching vibrations respectively. 1,535 cm^−1^ and 578 cm^−1^peaks are assigned to C=C and C-Cl vibrations.

**FIGURE 2:**
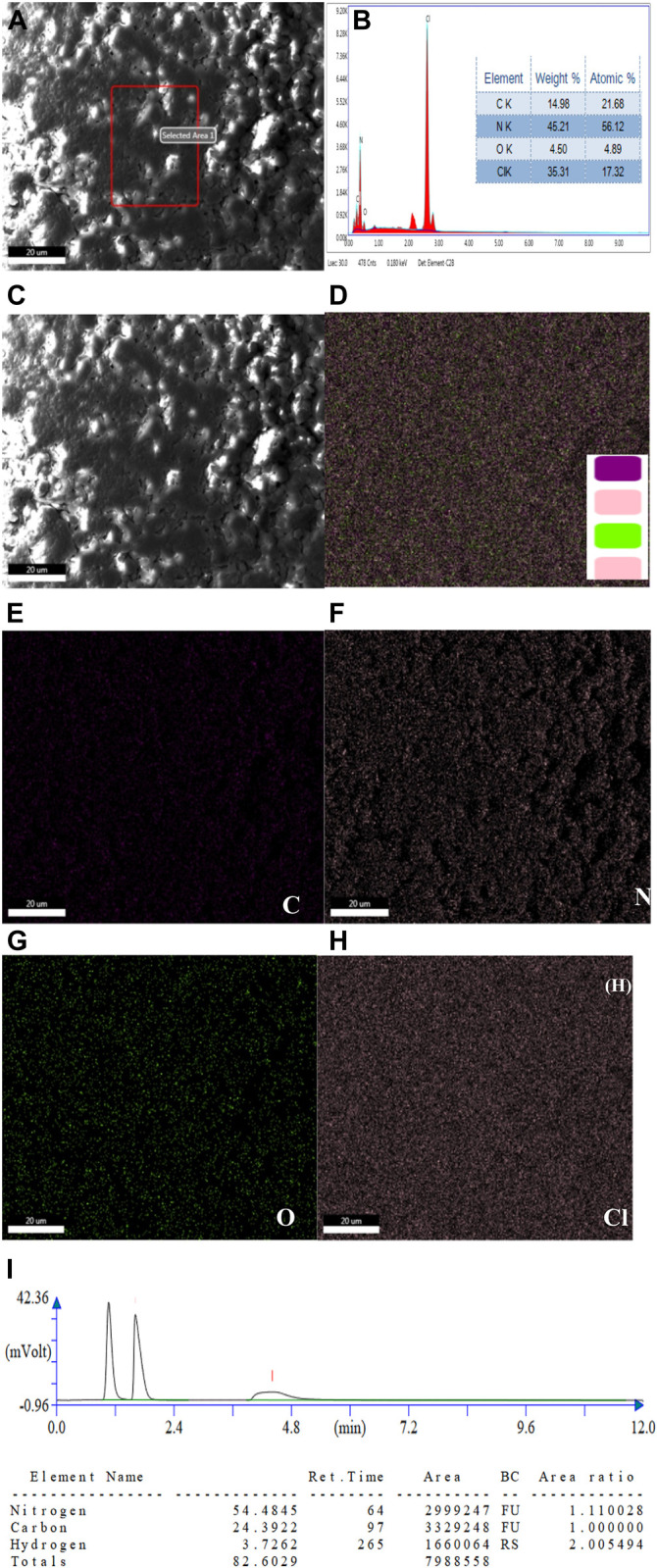
**(A)** FESEM image of Cl-CNDs with selected area for collecting the elements present **(B)** corresponding EDX spectrum along with their elemental composition table. **(C)** FESEM image of Cl-CNDs. **(D)** EDX elemental mapping of Cl-CNDs with their corresponding individual mapping **(E)** carbon **(F)** nitrogen **(G)** oxygen **(H)** chlorine. **(I)** Elemental analysis chromatogram with elemental composition table.

EDX analysis and elemental mapping were used to determine the composition of Cl-CNDs, as shown in Figure 2. A typical elemental composition of C = 14.98%, N = 45.21%, O = 4.50% and Cl = 35.31%, Figure 2B, is observed for the Cl-CNDs.The elemental mapping of the samples confirmed the homogeneity of the Cl-CNDs in terms of evenly distributed carbon, nitrogen, oxygen, and chlorine atoms, Figures 2C–H. The elemental composition of the Cl-CNDs determined by the elemental analyzer, Figure 2I, resulted in a composition of C = 24.39%, N = 54.48% and H = 3.72%, which closely matches the results from the EDX analysis.

**FIGURE 3:**
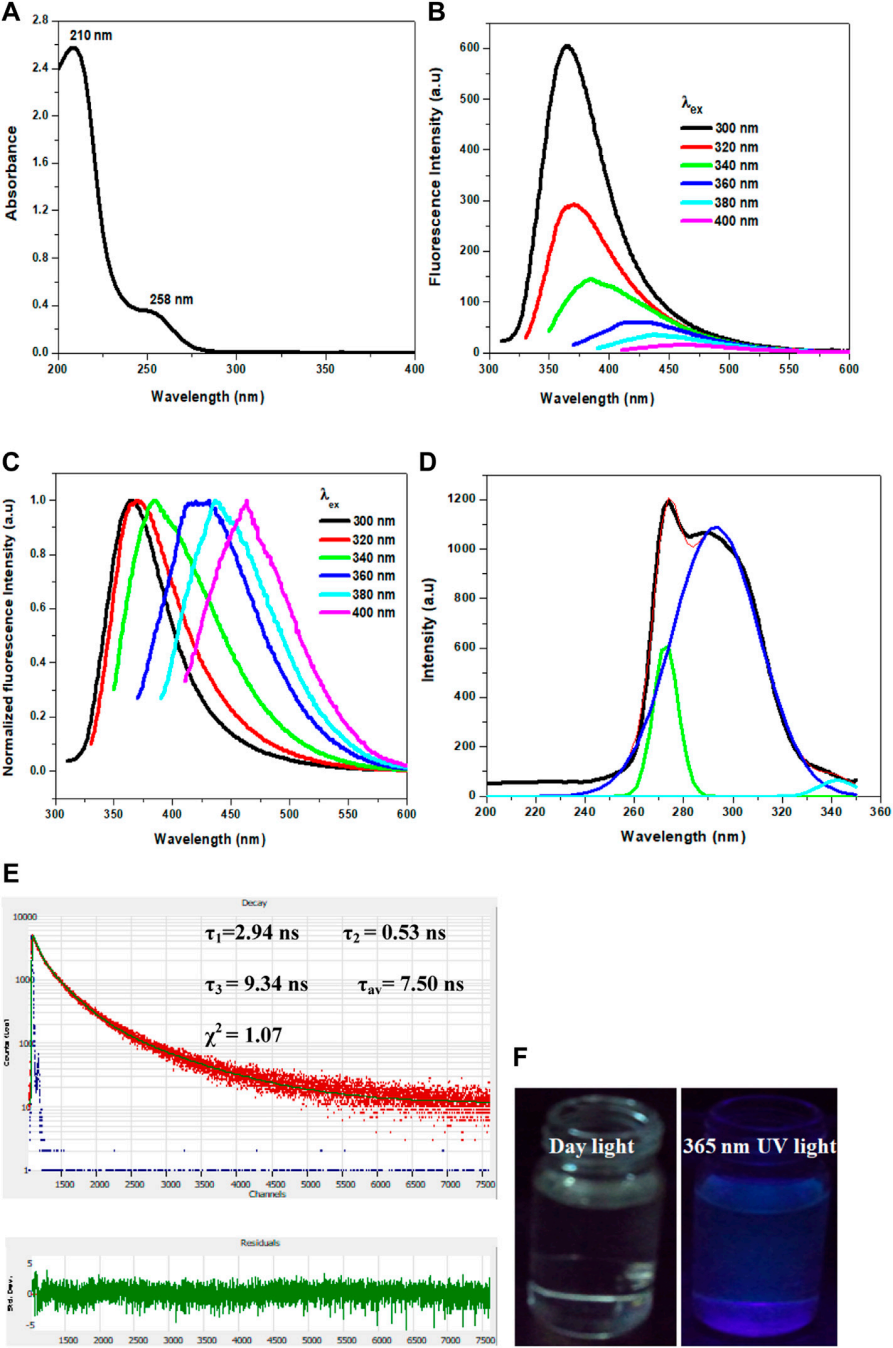
**(A)** UV-visible spectrum of Cl-CNDs in aqueous medium. **(B)** Fluorescence spectra of Cl-CNDs with variation of excitation wavelength from 300 to 400 nm in aqueous medium **(C)** corresponding normalized plot of **(B)**.**(D)** Fluorescence excitation spectra of Cl-CNDs monitored at 360 nm emission in aqueous medium with deconvolution showing the multiple peaks. **(E)** Time resolved fluorescence decay curve of Cl-CNDs with residual fitting. **(F)** Photograph of fluorescence of Cl-CNDs in aqueous dispersion under daylight and 365 nm UV light irradiation respectively.

The optical properties of Cl-CNDs were studied with UV-visible and fluorescence spectroscopy. The absorption peak at 210 nm was due to the π-π^*^ transition originating from an aromatic ring while the peak observed at 258 nm was assigned to the n-π^*^ transition, (Liu et al., 2011) Figure 3A. The aqueous dispersion of Cl-CNDs shows the excitation dependent shift of fluorescence emission maxima, indicating the presence of multiple emissive states. The fluorescence emission center of Cl-CNDs had a red shift from 360 to 460 nm on variation of the excitation wavelength from 300 to 400 nm, which is characteristic of carbon nitride nanoparticles (Ding et al., 2015; Patir and Gogoi, 2018), Figure 3B-C. The excitation spectrum of Cl-CNDs is recorded by monitoring the emission center at 360 nm, Figure 3D. This shows a broad spectrum that can be deconvoluted to three peaks at 270 nm, 295 nm, and 342 nm, which is indicative of there being at least three excitation centers involved in the emission process. The time resolved fluorescence decay curve of Cl-CNDs, Figure 3E, fits to three exponential models giving fluorescence life times of τ_1_ = 2.94 ns, τ_2_ = 0.53 ns, τ_3_ = 9.34 ns, with an average fluorescence life time of τ_av_ = 7.50 ns. These results further confirm the presence of multiple emitters. Commonly observed wavelength dependent red shift of emission spectrum in carbogenic nanoparticles has been attributed to diverse phenomena like quantum confinement, a single particle with multiple functional groups, the composition of individual emitters within a particle, and the ensemble of emissive states (Baker and Baker, 2010; Hsu and Chang, 2012, Dekaliuk et al., 2014, Khan et al., 2015, Demchenko, 2019). Here, the observation of excitation wavelength dependent fluorescence shift is assigned to the presence of multiple excitation centers, which are generated due to the difference in the local environment around individual emitters. These emission centers are excited at different excitation wavelengths and emit subsequently (Dekaliuk et al., 2014). Blue fluorescence is observed for the clear aqueous dispersion of Cl-CNDs under 365 nm UV irradiation, Figure 3F.

**FIGURE 4:**
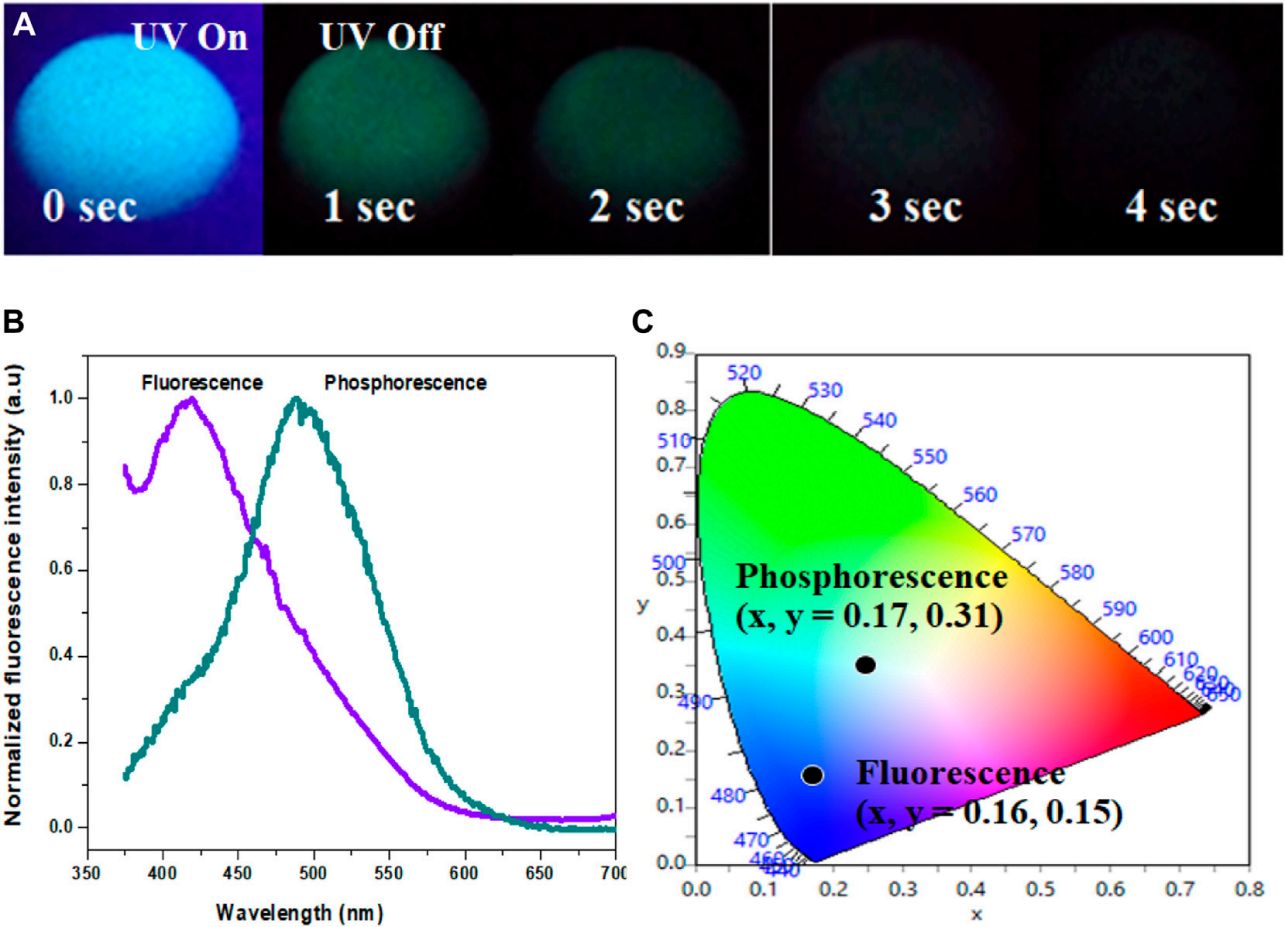
**(A)** Photograph showing luminescence (fluorescence and phsophorescence) of Cl-CNDs (solid state) under 365 nm UV light on and off. **(B**) Fluorescence and phosphorescence spectra of Cl-CNDs **(C)** corresponding CIE chromaticity diagram with color coordinates of **(B)**.

The Cl-CNDs in a solid state also show blue fluorescence under 365 nm UV light irradiation and green phosphorescence afterglow for a few seconds after the light is turned off, Figure 4A. The phosphorescence emission is centered at 490 nm while the fluorescence is at 419 nm, Figure 4B. The visual perception of the fluorescence and phosphorescence is obtained from the CIE chromaticity diagram, Figure 4C. The shift of fluorescence to phosphorescence emission centers is 71 nm, implying the difference between the singlet-triplet energy levels to be ∼0.48 eV (Deng et al., 2013).

**FIGURE 5:**
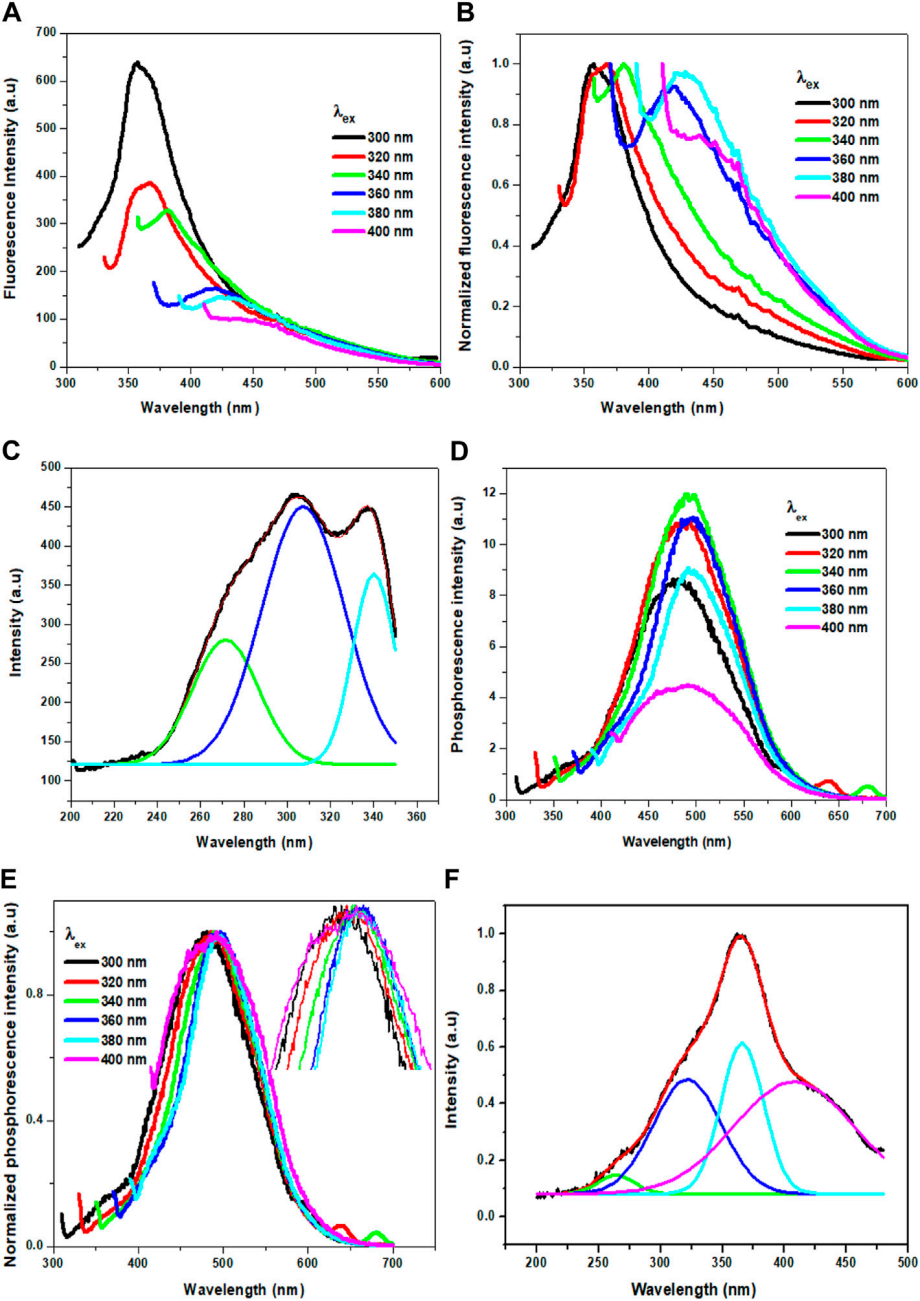
**(A)** Fluorescence of Cl-CNDs (solid state) with a variation of excitation wavelength from 300 to 400 nm **(B)** corresponding normalized spectra of **(A)**. **(C)** Fluorescence excitation spectra of Cl-CNDs monitored under 360 nm emission with deconvolution showing the multiple peaks. **(D)** Phosphorescence of Cl-CNDs with a variation of excitation wavelength from 300 to 400 nm **(E)** corresponding normalized spectra of **(D)** (inset: expansion of peaks to visualized the shifting). **(F)** Phosphorescence excitation spectra of Cl-CNDs monitored under 490 nm emission with deconvolution showing the multiple peaks.

Interestingly, the Cl-CNDs powder also shows an excitation wavelength dependent fluorescence similar to the aqueous solution, Figures 5A,B. With the variation of the excitation wavelength from 300 to 400 nm, the fluorescence intensity of Cl-CNDs powder decreases continuously with red shift from 355 to 450 nm. The fluorescence excitation spectrum of Cl-CNDs in a solid state can again be deconvoluted to bands at 270 nm, 302 nm, and 337 nm Figure 5C, indicating the presence of three excited energy states contributing to the fluorescence process. Compared with the excitation peaks of Cl-CNDs in liquid dispersion, as shown in Figure 3D, the excitation peaks at 270 nm, 295 nm, and 342 nm are at similar wavelengths in both solid and liquid phases. Thus the observation of similar excitation dependent red shift of emission maxima in liquid and solid phases rules out the ensemble of states generated by competitive solvent relaxation and fluorescence emission as the origin of the red shift observed. Rather, our experimental findings point towards the presence of emission centers in different local environments, leading to multiple excited states emitting at different wavelengths. Similarly, we have also recorded the phosphorescence spectra of the Cl-CNDs powder under different excitation wavelengths, Figures 5D,E. On increasing the excitation wavelength from 300 to 400 nm, the phosphorescence emission center is red-shifted from 480 to 490 nm with increasing phosphorescence intensity until 340 nm excitation wavelength, thereafter the phosphorescence intensity decreases till 400 nm with phosphorescence emission centre red shift from 490 to 500 nm (Li et al., 2019). Thus the excitation wavelength dependent red shift of phosphorescence emission maxima is a confirmation of the red edge effect due to the emission from the lowest triplet states of luminogens at different rigid polar environments rather than the emission from different triplet states of the same luminogen. Since the lifetime of the phosphorescence process is considerably longer, the possibility of emission from higher triplet states is negated. This is an indication that stabilization of the electronic states of Cl-CNDs in a solid state leads to the observation of phosphorescence.

The phosphorescence excitation spectrum of Cl-CNDs, Figure 5F, is deconvoluted to four peaks centered at 261, 318, 366, and 418 nm. This indicates that the excitation states contribute to the phosphorescence. Comparing Cl-CNDs phosphorescence excitation spectrum with the solid-state fluorescence excitation spectrum, as observed in Figure 5C, it can be said that the same states have contributed to the fluorescence and the phosphorescence process. Thus the fluorescence and the phosphorescence have the same origin: the Cl-CNDs. The shift of the lowest fluorescence excitation center 337 nm to the lowest phosphorescence excitation center 418 nm, is about 81 nm, suggesting their singlet-triplet energy gap to be ∼0.54 eV. The phosphorescence excitation spectrum overlaps with the UV-visible absorbance peak for C=O/N, Figure 3A, suggesting that the phosphorescence originates from the C=O/N groups of the Cl-CNDs (Li et al., 2016; Long et al., 2018). The C-Cl or C-NH_2_ groups present on the surface of the Cl-CNDs and provide rigidity via hydrogen bonding, stabilizing the triplet states, which in turn enhance phosphorescence. Furthermore, the hydrogen bonding of C-Cl or C-NH_2_ bonds of Cl-CNDs also prevents the oxygen or moisture-induced quenching of phosphorescence at room temperature. 

**FIGURE 6:**
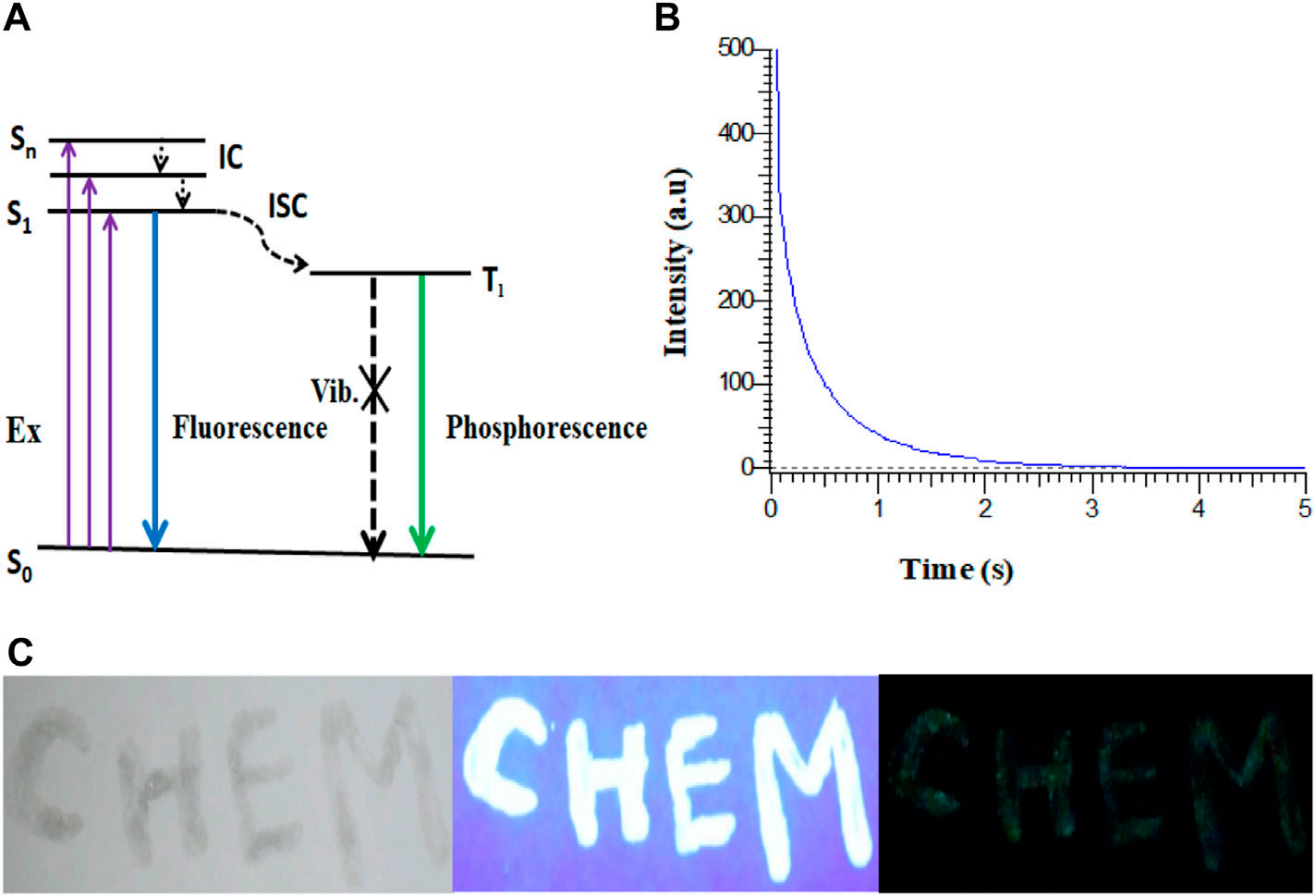
**(A)** Probable mechanism of RTP in Cl-CNDs. **(B)** Phosphorescence lifetime decay profile of Cl-CNDs under 360 nm excitation wavelength and emission monitored at 490 nm. **(C)** Application of Cl-CNDs as a phosphorescent ink for anti-counterfeiting (from left to right under daylight, 365 nm UV light on and off).

The probable mechanism of phosphorescence in Cl-CNDs is shown in Figure 6A. The electrons are raised from the ground state (S_o_) to higher excited states (S_n_) on absorption, followed by the electrons coming back to the lowest excited state (S_1_) by the internal conversion (IC) process. The S_1_ electrons are then transferred to the excited triplet state (T_1_) via the intersystem crossing process (ISC) from where electrons fall to S_o_, resulting in phosphorescence. The phosphorescence quantum yield of the Cl-CNDs was determined with the integrating sphere method and was found to be 2.32%, which is comparable to existing literature (Li et al., 2016; Long et al., 2018; Li et al., 2019).

The time resolved phosphorescence decay profile of the Cl-CNDs is measured under 360 nm excitation wavelength with emission monitored at 490 nm, as shown in Figure 6B. The phosphorescence lifetime of Cl-CNDs is found to be 657 ms. The phosphorescent Cl-CNDs are applied as a long afterglow ink. The Cl-CNDs powder is mixed with a transparent liquid paper gum and the characters ‘CHEM’ are written on non-reflecting filter paper, Figure 6C. These characters show fluorescence under 365 nm UV light irradiation and display green phosphorescence after the 365 nm UV light is turned off. The Cl-CNDs may find applications in different fields such as fluorescent sensors, phosphorescent glow-in-the-dark materials, and security encryption (Long et al., 2018; Wang et al., 2021).

## Conclusion

The present study presents a simple and cost-effective thermal strategy to synthesize an efficient matrix free carbon based phosphorescent nanomaterial using guanidine hydrochloride as the starting material. The obtained Cl-CNDs are highly miscible in an aqueous medium and display fluorescence and phosphorescence (solid state) under ambient conditions. The heteroatom bonds of the Cl-CNDs facilitate the effective intersystem crossing to triplet state from lowest singlet state, hence phosphorescence is observed. In addition, C-Cl or C-NH_2_ presence on Cl-CNDs inhibited the non-radiative decay from the triplet states as well as excluded the atmospheric oxygen via hydrogen bond formation. The average phosphorescence lifetime of Cl-CNDs is observed to be 657 ms. The phosphorescence quantum yield of Cl-CNDs is determined to be 2.32% by using the integrating sphere method. Moreover, Cl-CNDs are also demonstrated as phosphorescent ink on non-reflecting paper for the purpose of security marking. Hence, this work may help understanding and future applications of carbon nitride nanoparticles based on room temperature phosphorescent materials.

## Data Availability

The original contributions presented in the study are included in the article.
